# A low-cost virtual coach for 2D video-based compensation assessment of upper extremity rehabilitation exercises

**DOI:** 10.1186/s12984-022-01053-z

**Published:** 2022-07-28

**Authors:** Ana Rita Cóias, Min Hun Lee, Alexandre Bernardino

**Affiliations:** 1 Institute for Systems and Robotics, Instituto Superior Técnico, Universidade de Lisboa, Lisbon, Portugal; 2 Singapore Management University, Singapore, Singapore

**Keywords:** Upper extremity stroke rehabilitation therapy, Virtual coach, Compensation assessment, 2D video analysis

## Abstract

**Background:**

The increasing demands concerning stroke rehabilitation and in-home exercise promotion grew the need for affordable and accessible assistive systems to promote patients’ compliance in therapy. These assistive systems require quantitative methods to assess patients’ quality of movement and provide feedback on their performance. However, state-of-the-art quantitative assessment approaches require expensive motion-capture devices, which might be a barrier to the development of low-cost systems.

**Methods:**

In this work, we develop a low-cost virtual coach (VC) that requires only a laptop with a webcam to monitor three upper extremity rehabilitation exercises and provide real-time visual and audio feedback on compensatory motion patterns exclusively from image 2D positional data analysis. To assess compensation patterns quantitatively, we propose a Rule-based (RB) and a Neural Network (NN) based approaches. Using the dataset of 15 post-stroke patients, we evaluated these methods with *Leave-One-Subject-Out* (LOSO) and Leave-One-Exercise-Out (LOEO) cross-validation and the $F_1$ score that measures the accuracy (geometric mean of precision and recall) of a model to assess compensation motions. In addition, we conducted a pilot study with seven volunteers to evaluate system performance and usability.

**Results:**

For exercise 1, the RB approach assessed four compensation patterns with a $F_1$ score of $76.69 \%$. For exercises 2 and 3, the NN-based approach achieved a $F_1$ score of $72.56 \%$ and $79.87 \%$, respectively. Concerning the user study, they found that the system is enjoyable (hedonic value of 4.54/5) and relevant (utilitarian value of 4.86/5) for rehabilitation administration. Additionally, volunteers’ enjoyment and interest (Hedonic value perception) were correlated with their perceived VC performance ($\rho = 0.53$).

**Conclusions:**

The VC performs analysis on 2D videos from a built-in webcam of a laptop and accurately identifies compensatory movement patterns to provide corrective feedback. In addition, we discuss some findings concerning system performance and usability.

## Background

Post-stroke patients often suffer from physical impairment [1], with a weakened body side [2, 3], leaving them incapable of accomplishing daily tasks [4, 5]. Rehabilitation poses a crucial strategy to reduce stroke effects, prevent disability and stroke recurrence, demanding a lot of time investment [4–6]. However, the growing number of patients lead therapists to struggle in giving them the necessary attention and rehabilitation administration [5, 7]. Therefore, therapists frequently recommend the repetition of specific exercises [4, 6, 8] as in-home rehabilitation [9] to improve patients’ functional abilities. Nonetheless, patients have difficulty with keeping their motivation and engagement with in-home exercises without professional supervision. Low adherence and incorrect execution of in-home exercises negatively affect their recovery process [2, 10].

While exercising, patients regularly exhibit compensatory motions, using additional or new body joints, to aid in task accomplishment [3, 4, 11, 12]. The most typical compensation behaviors are trunk displacements, rotation, and shoulder elevation [3, 11]. As the persistence of compensatory movements may obstruct real motor function recovery, patients require exercise instructions and feedback to reduce these movement patterns [3, 11, 12].

The escalating demands towards in-home rehabilitation [1, 5] raised the need for quantitative measures to evaluate patients’ motor performance [9, 13]. Quantitative assessment allows tracking patients’ progress and the formulation of standard therapy regimens [9, 14]. Assistive systems with quantitative assessment, as Virtual coaches (VCs), can aid patients to perform in-home exercises [15, 16]. VCs must be adequate, affordable, and accessible, with an interaction model to keep the user engaged [15–17]. Also, they must evaluate patients’ performance to provide therapists with the required data to track their progress and support clinical decisions [9, 13].

Previous works investigated computer-based solutions for in-home upper extremity rehabilitation [17–19]. The proposed systems have complex interaction models which provide visual and audio feedback [17–19]. They utilize marker-based motion capture [19] or Kinect-based [17, 18] systems to assess patients’ exercise performance through motion kinematic analysis. Exercise instructions and feedback—such as error messages and direct performance ratings—are displayed on screens [19] and tablets [17] using graphical interfaces [18].

Researchers identified kinematic variables to characterize impaired motion patterns [9, 13, 14, 20, 21]. They provided automated methods to produce assessment scores highly correlated with Fugl-Meyer Assessment (FMA) scores, a conventional assessment test. Global performance scores provide patients with exercise ratings and therapists with clinically relevant information [9, 13, 20].

In addition, research teams conducted user studies with post-stroke patients to evaluate their systems’ impact on light supervised rehabilitation sessions [17–19]. They pointed out the importance of simple technical setups and reliable performance evaluation for in-home and independent use.

Although prior works [17–19] demonstrate the potential of computer-based systems to improve movement quality, their systems’ technical setups are still very complex for massive in-home use, involving several devices and objects. Quantitative assessment methods are based on 3D pose data kinematic analysis requiring specific motion capture devices for 3D data acquisition as Kinect. Such systems are less affordable and accessible and of complicated use, being less suitable for in-home therapy. With the investigation of novel means to assess patient’s performance from built-in cameras from tablets and laptops, systems would better fit in an affordable and accessible in-home therapy. However, there has been limited investigation on low-cost quantitative assessment methods to provide real-time feedback on compensation patterns.

In this work, we present a low-cost Virtual coach (VC) for stroke rehabilitation and a preliminary study to evaluate its usability. This VC is composed of a single laptop with a built-in webcam to monitor exercises of a user and provide real-time feedback on compensatory movements to assist user engagement in therapy. We present methods to assess quantitatively in real-time motor compensation from rehabilitation exercises through 2D video analysis. To enable real-time assessment, we labeled dataset videos frame-by-frame on compensation patterns. In addition, through an exploratory user study with seven volunteers, we collect some findings on VC usability.

## Virtual coach

We describe a Virtual coach (VC) that monitors upper extremity stroke rehabilitation exercises, assessing motor compensation behaviors. From the related work [15, 17–19] and therapists’ advice, we list a set of VC system requirements:

- Present an exercise demonstration;
- Display a patient’s image while exercising as if looking at a mirror;
- Provide clear audio instructions, cues for posture correction, encouragement, and suggest task repetition;
- Display visual markers indicating the arm target position and the existence of compensation.

Our VC is a *Reflex Agent*. It analyses body keypoints and quantitatively assesses patient’s exercises to update the *state*. Based on the user’s previous *state*, current *state* and a specified *time* interval, the agent selects an *action*. These actions include:

- Display of position markers—the rectangle indicating patient’s valid positioning;
- Display of the hand target marker;
- Display of compensation indicator markers—shoulder and trunk markers;
- Audio speech and respective subtitles—instructions, suggestions, encouragement, and praise.

Tables 1 and 2 describe the states and actions of the VC with their trigger rules, respectively.

**Table 1:** Space state of VC state transition

State space $\mathcal {S}$	Description
*Out* (*o*)	Patient not placed in the correct position
*In* (*i*)	Patient placed in the correct position
*Exercise* (*e*)	Exercise and movement trial beginning
*Normal* (*n*)	Normal movement pattern
$Trunk \text { } rotation$ (*tr*)	Patient rotates the torso
$Shoulder \text { } elevation$ (*se*)	Patient elevates the shoulder
$Trunk \text { } displacement$ (*td*)	Patient displaces the torso
*Target* (*tg*)	Patient reaches the target position

**Table 2:** Virtual coach actions related to state transitions and also permanence in the same state

State transition no.	Rules	Actions
1	$State_{prev} = o$ $State = o$ $Time>th_{pos}$	Patient not well-positioned: VC suggests body repositioning; position rectangle in red color.
2	$State_{prev} = \mathcal {S}/\{o,e,tg\}$ $State = o$	Patient moves away from correct position: VC suggests body re-positioning; position rectangle in red color.
3	$State_{prev} = o$ $State = i$	Patient well-positioned: position rectangle in green color; VC gives exercise directions.
4	$State_{prev} = i$ $State = e$	Exercise beginning: VC displays target position marker (green).
5	$State_{prev} = \mathcal {S}/\{o,i,tg\}$ $State = e$	Patients stops moving: VC proposes movement repetition.
6	$State_{prev} = e$ $State = n$	The VC starts evaluating patient’s performance and asks one to reach the target position.
7	$State_{prev} = \{tr,se,td,n\}$ $State = \{tr,se,td,n\}$ $Time>th_{tg}$	Patient takes too much time reaching the target position: VC encourages patient to reach the target.
8	$State_{prev} = \{tr,se,td,n\}$ $state = tg$	Patient reaches the target: VC praises the patient; target position marker in blue color.
9	$State_{prev} = \{tr,se,td,n\}$ $State = tr$	Patient describes trunk rotation: VC suggests posture correction; it displays trunk compensation marker (red).
10	$State_{prev} = \{tr,se,td,n\}$ $State = se$	Patient describes shoulder elevation: VC suggests correction; VC displays shoulder compensation marker (red).
11	$State_{prev} = \{tr,se,td,n\}$ $State = td$	Patient describes displaces the torso: VC suggests posture correction; VC displays trunk compensation marker (red).

### Compensation quantitative assessment methods

To assess different compensation patterns from 2D videos, we propose an approach composed of the following steps: *Body Keypoint Extraction* and *Selection*, *Data Normalization*, and *Classification*. We investigate two classification approaches—a Rule-based (RB), our baseline method, and a Neural Network (NN) based approach. As in previous works [9], we present a set of *Kinematic Variables*, revealing compensation description. Kinematic variables are given as features to the RB classifier. For the NN-based classifier, we provide normalized body keypoints as features. We represent these methods with the mathematical notation specified in Table 3.

**Table 3:** Mathematical notation

Equation	Description
$p_j^t = [ x_j^t y_j^t ]'$	$[ x_j^t y_j^t ]'$ denotes the transposed vector of 2D coordinates in the image of a body joint *j* from a set of joints *J* (Fig. 1); *t* denotes the frame number
$P^t(j_1,j_2) = p^t_{j_2} - p^t_{j_1} = [ x^t_{j_2} - x^t_{j_1} y^t_{j_2} - y^t_{j_1} ]'$	Vector directed from joint $j_1$ to joint $j_2$
$\Vert P^t(j_1,j_2)\Vert$ $d^t(j_1,j_2) = \Vert p_{j_1}^t - p_{j_2}^t \Vert$	$\Vert P^t(j_1,j_2)\Vert$ is the euclidean norm of vector $P^t(j_1,j_2)$ and, alternatively, $d^t(j_1,j_2)$ is the euclidean distance between two selected joints, $j_1$ and $j_2$
$\Delta x^t(j_1,j_2) = x^t_{j_1} - x^t_{j_2}$ $\Delta y^t(j_1,j_2) = y^t_{j_1} - y^t_{j_2}$	Displacement between two selected joints, $j_1$ and $j_2$, in the *X* ($\Delta x$) and *Y* ($\Delta y$) axis
$a^t(j_1,j_2,j_3) = \arccos \bigg ( \frac{P^t(j_2,j_1) \cdot P^t(j_2,j_3)}{\Vert P^t(j_2,j_1) \Vert \cdot \Vert P^t(j_2,j_3) \Vert } \bigg )$	Angle between two vectors, $P^t(j_2,j_1)$ and $P^t(j_2,j_3)$, defined by two points, $j_2$ to $j_1$ and $j_2$ to $j_3$

#### Body keypoints extraction and selection

**Fig. 1:**
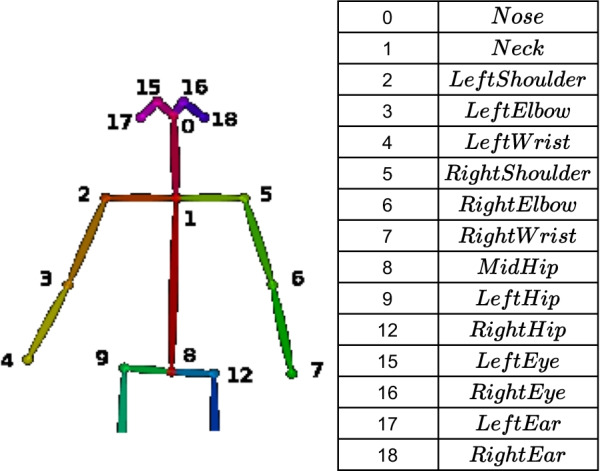
OpenPose Body keypoints

To extract the body joints’ 2D pose data, we use OpenPose [22], a software library that provides the 2D position of 25 body keypoints (body skeleton) in the image coordinate system, $\{I\}$ (Fig. 1). Each keypoint provided is denoted by $o^t_j = [ p^t_j \text { } s^t_j ]' = [ x^t_j \text { } y^t_j \text { } s^t_j ]'$. Here, $p^t_j = [ x^t_j \text { } y^t_j ]'$ denotes the 2D coordinates of a body keypoint *j*, *t* is the frame number, and $s^t_j$ is a confidence score of keypoint detection. Following [9], we selected the following keypoints to describe patients’ movements: *Nose*, *Eye*, *Neck*, *MidHip*, *Hip*, *Shoulder* *LeftEye*, *RightEye*, *RightHip*.

When selecting the most relevant keypoints to describe patients’ movements, we consider the three scenarios (S1, S2, and S3) concerning patient positioning in front of the camera: a patient facing the camera (S1) and with the affected arm facing the camera in a perpendicular (S2) and oblique (S3) positions. For S2 and S3, only the affected side is completely visible in the image.

#### Data transformation and normalization

In a real-world setting, patients have body parts of different sizes and occupy different locations regarding the camera. Accordingly, we perform keypoint normalization in three steps: transformation, normalization, and mirror. First, we apply rigid body transformation to overcome distinct patient positions. We transform each keypoint from the image coordinate system, $\{I\}$, to the body coordinate system, $\{B\}$, in which the patient’s joint *MidHip* ($j = 8$) is the origin.

Next, we normalize each keypoint coordinates in $\{B\}$ to the patient’s spine length, $d^1(p_{1}, p_{8})$, measured in $t=1$, to overcome distinct body part dimensions. Finally, for the NN-based approach, to give the healthy side as a reference, we mirror the joints to the *X* axis, in $\{B\}$, positive side. For the RB approach, the mirror step is not applied since each keypoint moves regarding another specified keypoint.

#### Kinematic variables

To assess compensation patterns from 2D body keypoints, we explore a set of measures for the three scenarios (S1, S2, and S3). From discussion with therapists, we identified four types of compensation: *Trunk Forward (TF)*, *Trunk Rotation (TR)*, *Shoulder Elevation (SE)*, and *Other (O)* trunk compensation patterns, such as trunk moving backward and trunk tilt. Given the compensation categories, Table 4 summarizes the respective kinematic variables.

**Table 4:** Kinematic variables

Scenario	Variable	Description
Trunk forward/backward		
S1	$\Delta H^t$	Observed changes in patient’s head position, detect through patient’s head area, $H^t$ ($t>1$)
S2 and S3	$a^t(p_8^1,p_1^1,p_1^t)$ $\wedge$ $\Delta x^t(p_1^t,p_1^1)$	Spine angular and linear displacements
Trunk rotation		
S1	$a^t(p^1_{2},p^1_{1},p^t_{2})$ $\wedge$ $a^t(p^1_{5},p^1_{1},p^t_{5})$	Simultaneous angular displacements of both shoulders
S2	$\Delta x^t(p^t_{2/5},p^t_{1})$	Shoulder displacement regarding joint 1 in ${}^BX$
S3	$| \Delta d^t(p^t_{2},p^t_{5})|$	Absolute changes in the observed chest length
Shoulder elevation		
S1	$a^t(p^1_{2/5},p^1_{1},p^t_{2/5})$	Shoulder elevation angle
S2 and S3	$\Delta y^t(p^t_{2/5},p^t_{1})$	Shoulder displacement regarding joint 1 in *Y*
Trunk tilt		
S1	$a^t(p^1_{8},p^1_{1},p^t_{1})$	Spine angular displacement
S2 and S3	$| \Delta H^t|$	Absolute changes in patient’s head size

#### Classification approaches

As we intend to identify multiple compensation patterns from video frames, we deal with a Multilabel Classification (MLC) problem. We propose two classification approaches: a Rule-based (RB) and a Neural Network (NN) based. In RB classification models, a set of *if-then rules* is applied to a collection of features to provide a predicted label [23]. We apply a set of independent rules to each kinematic variable from Table 4 to assess each compensation category, shown in Table 5 for each scenario (S1, S2, and S3). Table 5 details that a rule *r* (e.g., $r=SE$ denotes Shoulder Elevation) predicts a label, ${\hat{Y}}_r$, when a feature or set of features, $X_r$, obey a certain threshold value $th_r$, which limits the compensation pattern existence. Otherwise, the movement pattern is classified as Normal (${\hat{Y}}=4$). Additionally, multiple labels might be active (i.e., more than one compensation pattern happening simultaneously).

**Table 5:** Rules of the RB classification method to determine the different categories of compensation: Trunk Forward (TF), $Y=0$; Trunk Rotation (TR), $Y=1$; Shoulder Elevation (SE), $Y=2$; Other (O), $Y=3$. For normal movements $Y=4$

Scenario	Rules
Trunk forward (TF)/Trunk backward (O)	
S1	$${\hat{Y}} = {\left\{ \begin{array}{ll} 0 &{} \text {if } \, \Delta H^t > th_{TF} \\ 3 &{} \text {if } \, \Delta H^t < -th_{TF} \\ 4 &{} otherwise \end{array}\right. }$$
S2 and S3	$${\hat Y = \left\{ {\begin{array}{*{20}{ll}} 0& {\text{if }}\, {a^t}\left( {p_8^1,p_1^1,p_1^t} \right) > t{h_{TF}}\\ & \quad \wedge \Delta {x^t}\left( {p_{2/5}^t,p_1^t} \right) > 0 \\ \\ 3& {\text{if }}\, {a^t}\left( {p_8^1,p_1^1,p_1^t} \right) > t{h_{TF}}\\ & \quad \wedge \Delta {x^t}\left( {p_{2/5}^t,p_1^t} \right) < 0 \\ \\ 4& {otherwise} \end{array}} \right. }$$
Trunk rotation (TR) and Shoulder elevation (SE)	
S1	$$\hat Y = \left\{ {\begin{array}{*{20}{ll}} 2& {\text{if}}\, (1){\text{ }}{a^t}(p_{2/5}^1,p_1^1,p_{2/5}^t) > t{h_{SE}}\\ & \quad \wedge (2){\text{ }}{a^t}(p_{5/2}^1,p_1^1,p_{5/2}^t) < t{h_{SE}} \\ 1&{\text{if}}\, (1) > t{h_{SE}} \\ & \quad \wedge (2) > t{h_{SE}} \wedge (1) - (2) \approx 0 \\ \\ {1 \wedge 2} &{\text{if}}\, (1) > t{h_{SE}} \\ & \quad \wedge (2) > t{h_{SE}} \wedge (1) - (2) \gg 0 \\ \\ 4&{{\text{otherwise}}} \end{array}} \right.$$
Trunk rotation (TR)	
S2	$${\hat{Y}} = {\left\{ \begin{array}{ll} 1 &{} \text {if } \Delta x^t(p^t_{2/5},p^t_{1}) > th_{TR} \\ 4 &{} otherwise \end{array}\right. }$$
S3	$${\hat{Y}} = {\left\{ \begin{array}{ll} 1 &{} \text {if } |\Delta d^t(p^t_{2},p^t_{5})| > th_{TR} \\ 4 &{} otherwise \end{array}\right. }$$
Shoulder elevation (SE)	
S2 and S3	$${\hat{Y}} = {\left\{ \begin{array}{ll} 2 &{} \text {if } \Delta y^t(p^t_{2/5},p^t_{1}) > th_{SE} \\ 4 &{} otherwise \end{array}\right. }$$
Trunk tilt (O)	
S1	$${\hat{Y}} = {\left\{ \begin{array}{ll} 3 &{} \text {if } a^t(p^1_{8},p^1_{1},p^t_{1}) > th_{O} \\ 4 &{} otherwise \end{array}\right. }$$
S2 and S3	$${\hat{Y}} = {\left\{ \begin{array}{ll} 3 &{} \text {if } |\Delta H^t | > th_{TI} \\ 4 &{} otherwise \end{array}\right. }$$

As an RB model has the advantage of easy comprehension [23–25], our VC utilizes this method to determine when a user performs compensation. Additionally, we can change rules’ threshold values $th_r$ (Table 5) adjusting compensation assessment detection sensitivity.

While dealing with an MLC problem, we consider two situations: multiple label occurrence and label imbalance (labels more frequent than others). We apply binarization technique/one-hot encoding to the set of labels assigned to each frame (i.e., a vector of 0 s and 1 s, with 1 encoding the active labels) [25]. Then, we apply *One-vs-Rest*, training a classifier for each label against all others [26] so that one label prediction does not influence the other. The model generates predictions on each label, which are then combined to produce a multilabel response.

**Fig. 2:**
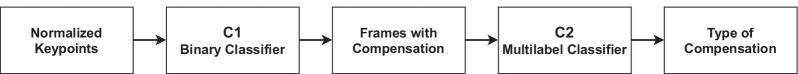
NN-based approach to assess compensation patterns

For the NN-based approach, our classifier must be robust enough to not assign a label to a frame denoting compensation and indicate good movement quality (*Normal* movement patterns, i.e., without compensation). Also, we have a much higher number of samples considered *Normal* than frames corresponding to each compensation category. Thus, we divide our problem into two problems, a binary and a multilabel. First, a binary classifier (C1) determines compensation existence. Second, a multilabel classifier (C2) concludes the described compensation patterns from the frames with compensation detected by C1. Figure 2 represents our proposed approach.

### User interface

**Fig. 3:**
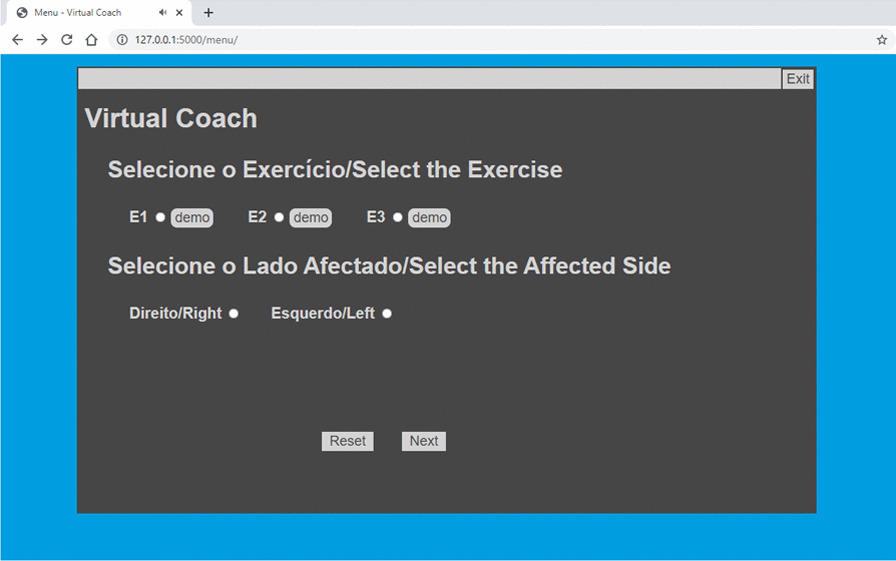
Virtual coach *Menu* web page

**Fig. 4:**
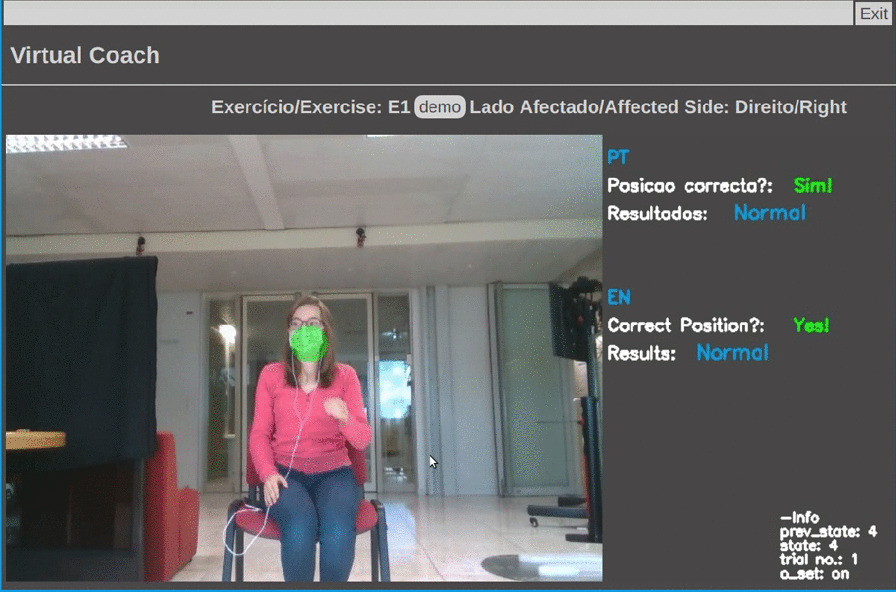
Virtual coach *Main* web page—display E1 target position

**Fig. 5:**
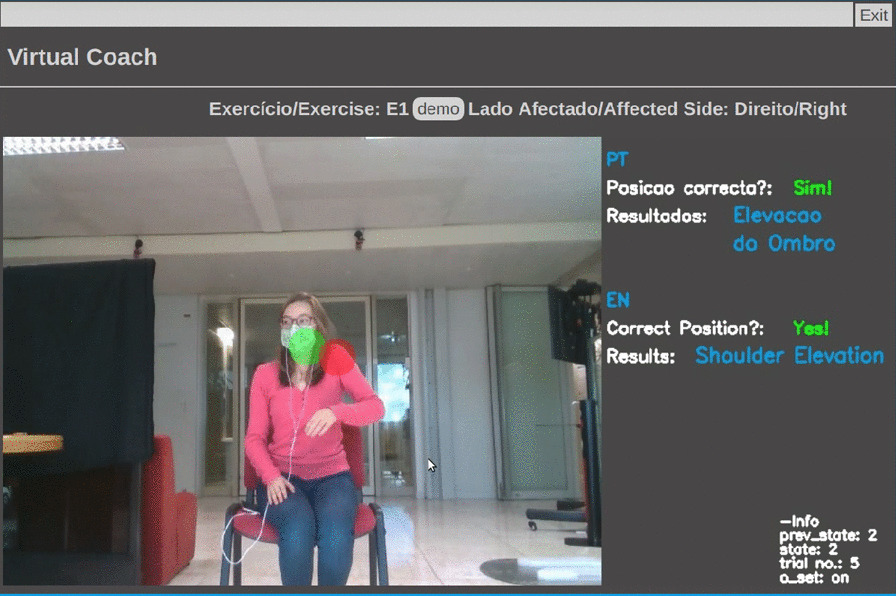
Virtual coach *Main* web page—shoulder elevation in E1 and display shoulder compensation marker

To establish an interaction with the user, we developed a web-based UI using Flask framework [27]. The UI is composed of four web pages: *Init*, *Menu* for exercise selection (Fig. 3), *Demo* (exercise demonstration), and *Main* (Figs. 4 and 5), in which the patient exercises and interacts with the VC. The main processing to track patient’s movements (keypoint extraction and compensation assessment) is handled in a remote server, accessed via WiFi, for faster processing and result extraction.

Once the user chooses an exercise, the user can watch each exercise demonstration. The VC describes three exercises (Table 6) and monitors user compensation behaviors during their execution. First, the VC verifies if the patient is correctly positioned to enable motion capture. Once the user is well placed, the VC gives exercise instructions, displays visual markers identifying the target position of an exercise (Fig. 4), and starts evaluating user movements. When the patient exhibits compensation, the VC suggests posture correction and displays a marker highlighting this behavior (Fig. 5). It also praises the user when one reaches the target position and encourages movement repetition.

**Table 6:** The three upper extremity exercises, E1, E2, and E3. Patients’ positioning scenarios and percentage of multi-labeled frames for each exercise

Upper extremity exercises		Positioning scenario	$1-P_{min}$
E1	*‘Bring a Cup to the Mouth’*	S1	$16.17 \%$
E2	*‘Switch a Light On’*	S1	$8.6 \%$
E3	*‘Move a Cane Forward’*	S2 and S3	$1.85 \%$

## Experiments

### Compensation quantitative assessment methods

#### The upper extremity rehabilitation dataset

**Fig. 6:**
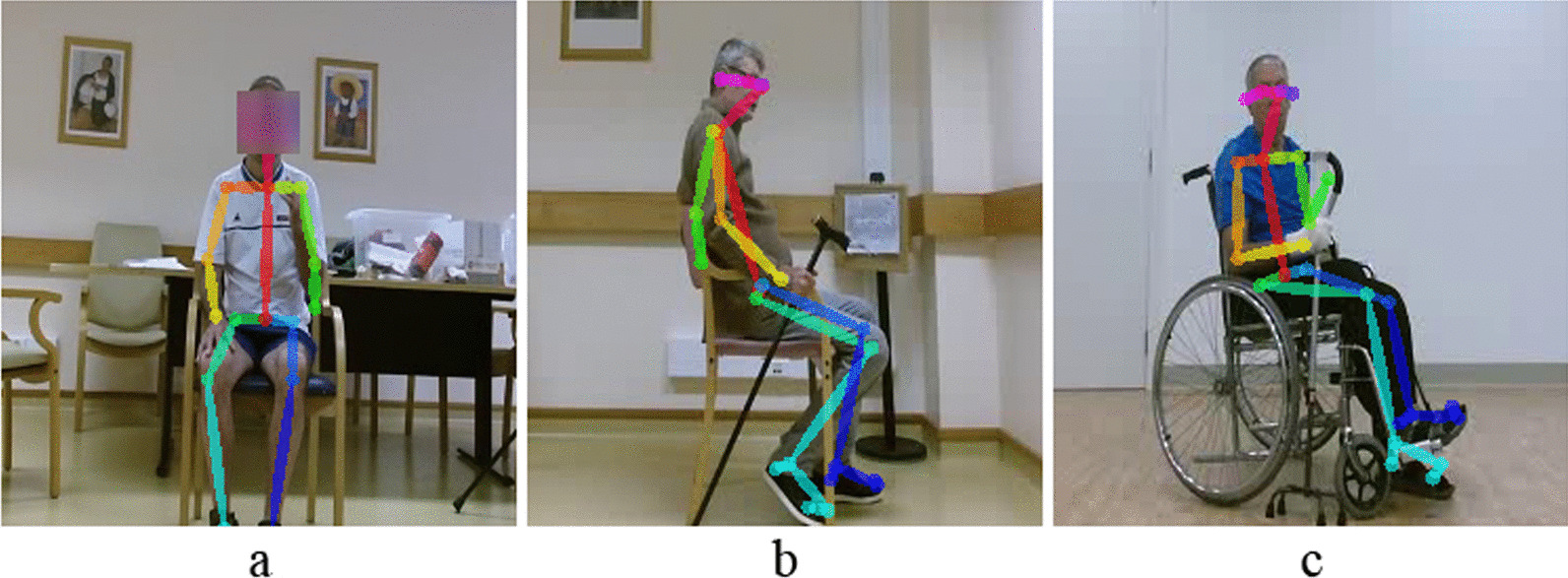
Examples of post-stroke patients performing exercises E1 and E3. E1 corresponds to S1 positioning scenario (**a**). In E3, patients are positioned according to S2 (**b**) and S3 (**c**) scenarios

This research uses the dataset from Lee et al. [9] work for the development and validation of proposed compensation assessment methods. It is a dataset of videos of 15 post-stroke patients performing three upper extremity exercises introduced in Table 6. The post-stroke profiles and respective Fugl-Meyer Assessment scores are presented in [9]. In exercise 1 (E1), the patient simulates holding a cup and brings the hand to the mouth as drinking. In exercise 2 (E2), the patient behaves as turning on a light switch. In exercise 3 (E3), the patient moves a cane forward and then back to its initial position.Post-stroke patients with an average age of $63 \pm 11.43$ years old [9] performed an average of 10 movement trials per exercise. Table 6 relates each exercise and positioning scenario (S1, S2, and S3). Figure 6 shows examples from the dataset of E1 and E3 exercises.

#### Data labeling process

Our work explores the following four compensation categories. We specified a set of labels, *Y*, denoting each one—i.e., for *Trunk Forward*, $Y = 0$; *Trunk Rotation*, $Y = 1$; for *Shoulder Elevation*, $Y = 2$; for *Other* patterns, $Y = 3$; and for *Normal* movements, $Y = 4$. Label $Y = 4$ denotes *Normal* movement patterns, i.e., without compensation. We labeled all frames of each video in agreement with Physical and Occupational therapists’ advice. We assigned one or more labels to each frame according to the visible compensation patterns.

#### Dataset cleansing

**Fig. 7:**
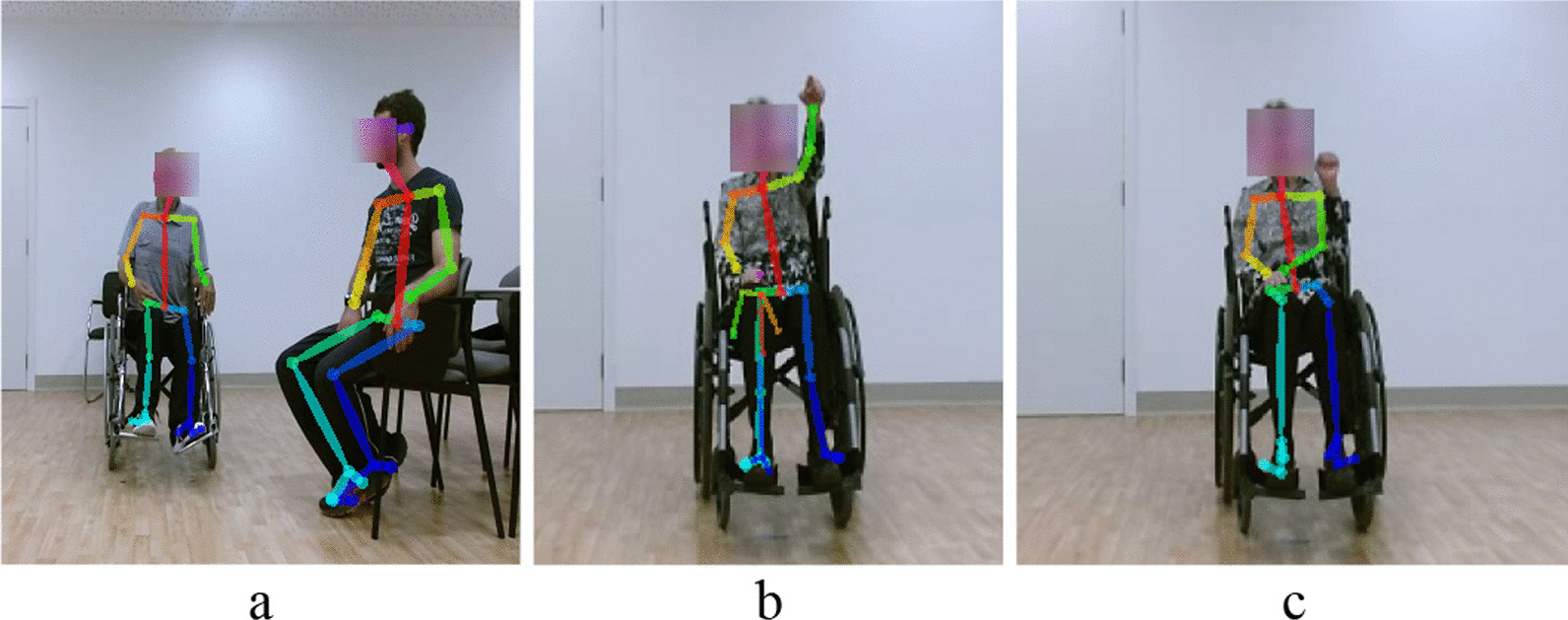
OpenPose extra person (**a**) and incorrect keypoint detection, e.g., extra skeleton (**b**) and keypoint misdetection (**c**)

Once we have the body keypoints extracted with OpenPose, it is crucial to consider three distinct situations concerning body skeleton detection: the presence of other people in the image beside the patient, extra skeletons, which do not necessarily belong to a person, and body keypoint misdetection (Fig. 7).

Considering a multi-person setting (e.g., the patient with a caregiver), the patient under evaluation is the closest person to the center of the image, measured by the distance to the image center, $d(p_{8},c_i)$.

Extra skeletons often do not have spine joints (*Nose*, *Neck*, and *MidHip*). Therefore, their confidence score, $s^t_j$, is zero. Thus, we removed these skeletons.

For keypoint misdetection, we consider a relevant body keypoint (affected side and opposite shoulder) was well detected if it has a confidence score higher than a specified value ($s^t_j > 0.36$). The remaining joints must have $s^t_j > 0$. We removed every video frame with body keypoints not meeting these conditions. In the case of frames with mispositioned body keypoints, with a detection confidence score of $s^t_j > 0.36$, we corrected keypoints’ coordinates using the MATLAB *imshow* function, which enables to access the coordinates of every point in the image.

#### Multilabel dataset characteristics

Our Multilabel Dataset (MLD) is a set of keypoints, from each video frame (sample), with one or more labels assigned denoting the compensation patterns of post-stroke patients. Before developing our classification models, we explore our MLD characteristics with two metrics: $1-P_{min}$ and *IRLbl*. Metric $P_{min}$ is the percentage of data samples with only one label active. Inversely, $1-P_{min}$ corresponds to the percentage of samples with more than one label assigned. As shown in Table 6, the dataset is almost single labeled, i.e., it has a low percentage of multi-labeled frames (frames with multiple compensation behaviors). Regarding label imbalance, the *IRLbl* metric shows the ratio between the occurrences of the most frequent label and each label [25]. Table 7 shows that, for the three exercises, label $Y=4$ is the most frequent, $IRLbl = 1$. For E1 and E2, $Y=1$ is poorly represented, $IRLbl \gg 1$, with only one patient exhibiting this compensation pattern. For E3, the less representative label is $Y=2$.

**Table 7:** Labels for each compensation and normal movements patterns and *IRLbl* metric for each one, for each exercise (E1, E2, and E3)

Compensation/Normal Pattern	Label	*IRLbl*		
		E1	E2	E3
Trunk forward	$Y=0$	–	–	3.54
Trunk rotation	$Y=1$	16.23	19.25	–
Shoulder elevation	$Y=2$	2.15	3.03	15.77
Other	$Y=3$	4.93	5.55	–
Normal	$Y=4$	1	1	1

#### Validation of kinematic variables for a rule-based approach

The validation of kinematic variables is crucial to determine the most suitable threshold values for the RB method and assess its efficiency in assessing compensation. We obtained the thresholds, $th_r$, through an error and trial methodology by observing the kinematic variables as a starting point. In the following figures, we observe the trajectories of kinematic variables over time. We filtered the keypoints signal (joints’ position over time) with a moving average filter (filtered signal) with a window of five frames as in [9] to reduce noise.

**Fig. 8:**
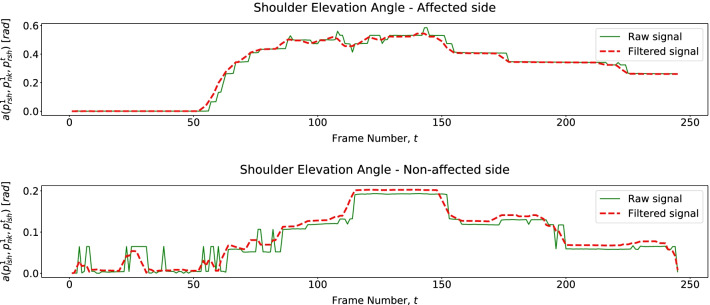
Patient shoulders’ elevation angles over time describing Trunk Rotation for E1

**Fig. 9:**
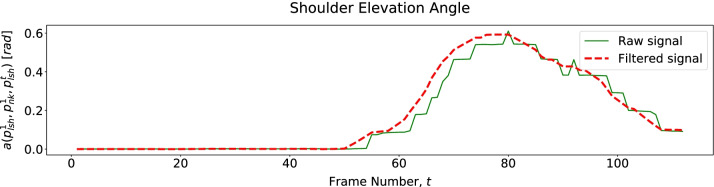
Patient affected shoulder elevation angle revealing Shoulder Elevation for E2

**Fig. 10:**
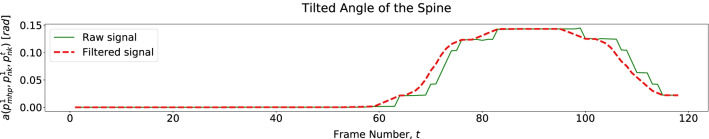
Patient tilted angle of the torso describing a trunk tilt (Other) for E2

**Fig. 11:**
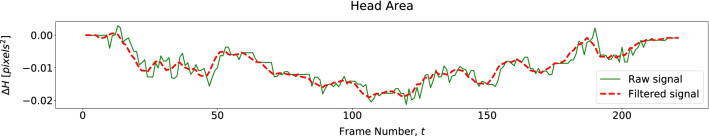
Head area over time, revealing trunk moving backward (Other) observed in the dataset for E2

Figure 8 shows we can assess trunk rotation from 2D pose data by tracking both shoulders (affected and unaffected) angular behavior as we hypothesized in Table 4. Trajectories of both shoulders reveal elevation (affected side) and decay (unaffected side) during trunk rotation simultaneously. This shoulder behavior is valid for both exercises E1 and E2. Also, for these exercises, as in previous works [9], we assess shoulder elevation and trunk tilt (Other compensation patterns) through affected shoulder and trunk angular displacement, respectively (Figs. 9 and 10). To evaluate trunk moving backward (Other) from 2D data, we assess variations in patients head area, $\Delta H$. Figure 11 shows that when a patient moves backward, $\Delta H$ decreases as hypothesized.

**Fig. 12:**
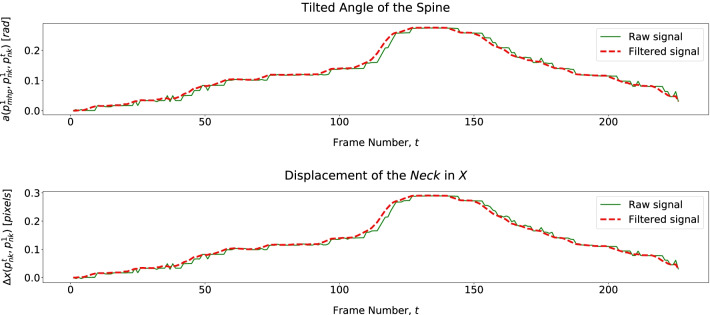
Patient tilted and of the spine and neck displacement over time, describing Trunk Forward in E3

**Fig. 13:**
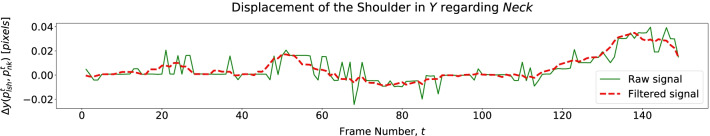
Patient shoulder displacement over time, describing Shoulder Elevation in E3

For exercise E3, we assess the torso moving forward through its linear and angular displacements (Table 4) described in Fig. 12. Since we only have 2D pose data, we assess shoulder elevation through its displacement regarding the *Neck* joint ($j = 1$). Figure 13 shows that a patient elevates the shoulder mainly when moving the cane back to its initial position.

#### Neural network based approach

We explore model architectures (i.e., one to three layers with 16, 24, 32, 48, 64, 96, 128, 192, 256, 384, and 512 hidden units) for a binary classifier (C1) and a multilabel classifier (C2) for the NN-based classification approach and with adaptive learning rate with several values for the initial learning rates (i.e., 0.0001, 0.0005, 0.001, 0.005, 0.01, 0.05, and 0.1). We adopt ‘*Adam Optimizer*’ with a mini-batch size of 5 and a maximum of 550 iterations. For C1 we apply ‘*ReLu*’ activation function and for C2 ‘*tanh*’ activation function. We implement C1 and C2 using the ‘*Scikit-learn*’ Python library [26].

#### Evaluation metrics and validation method

We use a set of metrics appropriated to an MLC problem to evaluate our classification models’ performance. We need metrics describing that a multilabel output result might be completely correct, partially correct, or incorrect [25]. We use *Precision*, *Recall*, $F_1$ score, and *HammingLoss* [25, 26]. We calculate the first four according to a *micro-averaging* strategy that joins the counters of correct and incorrect predictions and then calculates the metric. This way, rare labels are diluted between the most frequent labels [25, 26].

Metric *Precision* is the percentage of predicted labels truly significant for the sample. *Recall* expresses the classifier’s ability to detect all positive samples. Score $F_1$ is a weighted harmonic mean *Precision* and *Recall*, which measures classification accuracy. *HammingLoss* reveals the portion of mispredicted labels.

We resort to *cross-validation* to evaluate our models’ predictive ability and ensure generalization. Cross-validation consists of partitioning the dataset into small subsets. In the validation loop, all the sets except one are used for training, and the remaining set is used for validation [26, 28]. In the end, the performance measure determined in each loop is averaged.

First, we apply *Leave-One-Subject-Out* (LOSO) cross-validation since all patients in a post-stroke status have their specific motion pattern, Validating the models on each patient compensation pattern enables a better understanding of their classification performance and generalization capacity. Additionally, to verify model generalization to different exercises, we apply *Leave-One-Exercise-Out* (LOEO) cross-validation to the NN-based approach with exercises E1 and E2, in which patients have similar positioning during data collection.

### User study on the virtual coach

To achieve a preliminary evaluation of the VC usability, we performed experiments with a group of volunteers. We aim to investigate users’ perceptions of the VC on four dimensions: its Hedonic (H) value (i.e., users’ motivation and enjoyment while exercising and interacting with the VC), Utilitarian (U) value (i.e., users’ perception of the gains of exercising autonomously with the VC for post-stroke patients), System’s Performance (SP) (i.e., users’ perception of the VC’s accuracy on detecting compensation and correct feedback), and the Use Intention (IU) of VC users. In this study, we explore the following hypotheses:

1. Hedonic value perceptions are affected by the perception of Virtual Coach performance on monitoring exercise performance, detecting compensation, and by its interactive features;
2. There is a disparity on the VC perception between:
  - Post-stroke volunteer and non-stroke affected volunteers;
  - Older adults and younger adults mainly concerning VC **U** value.

Data collection and storage is in agreement with the General Data Protection Regulation (GDPR). To ensure these conditions, the Instituto Superior Técnico Ethics Committee reviewed and approved our experimental protocol.

#### Volunteers

We recruited seven volunteers to exercise their limbs with our system. When selecting the participants, we aimed to gather a diverse group concerning age, sex, and experience with the stroke thematic. Volunteers signed an Informed Consent authorizing the recording of their image necessary to the normal system operation. Table 8 presents the volunteers’ profiles and general information. The post-stroke volunteer has difficulty performing specific tasks (e.g., writing). Yet, he is fully recovered and does not perform compensatory movements.

**Table 8:** Profiles of the volunteers. General information: a Knows what (a) stroke is (b) Had a stroke (c) Some relative or close friend had a stroke (d) Followed the rehabilitation process closely

VID	Age	Sex	ND/A side	(a)	(b)	(c)	(d)
V01	25–34	M	Left	Y	Y	Y	Y
V02	55–64	F	Left	Y	N	Y	Y
V03	65–74	F	Left	Y	N	Y	Y
V04	65–74	M	Left	Y	N	Y	Y
V05	25–34	M	Left	Y	N	Y	N
V06	55–64	M	Left	Y	N	Y	N
V07	25–34	F	Left	Y	N	N	N

*VID*-volunteer ID, *ND*-non-dominant, *A*-affected, *F*-female, *M*-male, *Y*-yes, *N*-no

#### Experimental setup

Motivated to provide an affordable and accessible solution with a simple technical infrastructure, we only use a laptop with 6GB RAM and i5-4210U 2.40 GHz 2 Cores CPU with a built-in webcam in this experiment. We use the RB classification algorithm to assess compensation, which enables easy result interpretation and the adjustment of rules’ threshold values if necessary. The sessions took place in a domestic environment spacious enough to assure the placement of the laptop from the volunteer had a distance of $\approx 2.5 \text { } m$ to capture the participant’s relevant body joints.

#### Experimental procedure

At the beginning of a session, the researcher placed the laptop on a table or other support, giving the volunteer the possibility to be in front of the system exercising with enough space. She introduced the study, the entire procedure, and the functionalities of the UI. The volunteers were asked to perform the three exercises (E1, E2, and E3) with the arm from their affected side due to stroke or non-dominant body side. The researcher instructed volunteers to simulate the different compensation strategies while exercising. Volunteers repeated the movements at least five times. During the exercise, volunteers followed the VC instructions, and the researcher intervened when necessary. In the end, each participant answered a questionnaire giving their feedback about the VC. The session did not exceed 30 min.

#### Questionnaires

We collected both quantitative and qualitative responses from study participants evaluating the VC on each dimension (*H*, *U*, *SP*, and *IU*). We collect responses on volunteers’ enjoyment, motivation, and interest during the exercise session with the VC (*H* value). The VC’s benefits to health, aid on physical condition improvement, utility in autonomous exercises, and as a support to diminish struggles concerning rehabilitation administration (*U* value). Volunteers answered questions concerning their willingness to use the system (*IU*) and system effectiveness and reliability (*SP*).

The volunteers responded to each question on a 5-point Likert scale (quantitative)—from *‘1 = Strongly Disagree’* to *‘5 = Strongly Agree’*. In addition, we asked a follow-up question to gather more information about their responses.

## Results

### Compensation assessment results

Table 9 presents the evaluation metrics for the two proposed compensation assessment approaches,RB and NN-based, over three exercises (E1, E2, and E3). We describe the hyperparameters for the NN-based approach in Table 10. For E1, the RB classifier performed better than the NN-based classifier with an $F_1$ score of $76\%$. For E2 and E3, the NN-based classifier had a better performance than the RB approach with $F_1 = 73\%$ and $F_1 = 80\%$, respectively. Later, we discuss the differences in performance observed for the two approaches. In addition, LOEO cross-validation for the NN-based approach with E1 and E2, the classifier detects compensation with an $F_1$ score of $80\%$.

**Table 9:** Average results and standard deviation for the Rule-based (RB) and Neural Network (NN) methods for each exercise (E1, E2, and E3) with LOSO and LOES cross-validation

	Precision	Recall	$F_1 score$	Hamming loss
Rule-based (RB) Approach				
*Leave-One-Subject-Out* (*LOSO*) cross-validation				
$\text {E1}$	**0.75 ± 0.14**	**0.78** ± **0.12**	**0.76** ± **0.12**	**0.11** ± **0.07**
$\text {E2}$	$0.54 \pm 0.17$	$0.65 \pm 0.17$	$0.59 \pm 0.16$	$0.20 \pm 0.08$
$\text {E3}$	$0.69 \pm 0.27$	$0.71 \pm 0.26$	$0.70 \pm 0.26$	**0.13** ± **0.11**
Neural Network (NN) based Approach				
*Leave-One-Subject-Out* (*LOSO*) cross-validation				
$\text {E1}$	$0.71 \pm 0.23$	$0.70 \pm 0.25$	$0.70 \pm 0.24$	$0.18 \pm 0.15$
$\text {E2}$	**0.73** ± **0.21**	**0.73** ± **0.19**	**0.73** ± **0.19**	**0.15** ± **0.11**
$\text {E3}$	**0.80** ± **0.22**	**0.80** ± **0.21**	**0.80** ± **0.22**	$0.14 \pm 0.14$
*Leave-One-Exercise-Out* (*LOEO*) cross-validation with E1 and E2				
	$0.78 \pm 0.05$	$0.81 \pm 0.01$	$0.80 \pm 0.02$	$0.12 \pm 0.01$

The results in bold correspond to the best classifiers' performance for the different metrics for each exercise

*F*_1_ score is a measure of accuracy

**Table 10:** NN based approach classifiers’ hyperparameters

	#layers	#units/layer	Learning Rate${}_{init}$
C1			
E1	1	16	0.001
E2	2	16	0.001
E3	1	96	0.01
C2			
E1	1	64	0.001
E2	1	16	0.01
E3	1	16	0.001

### Virtual coach validation results

**Fig. 14:**
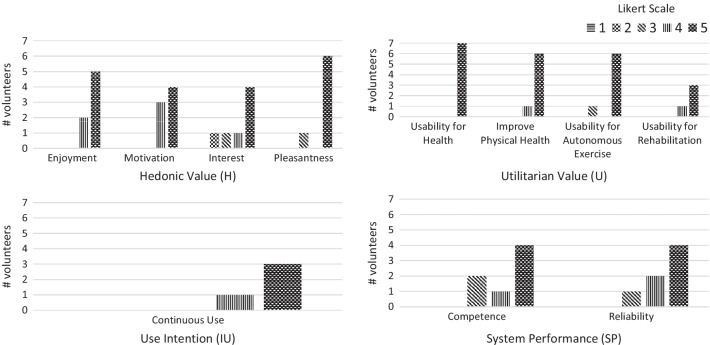
Perceptions of the Virtual coach on four dimensions: Hedonic Value, Utilitarian Value, Use Intention, and System Performance. Only volunteers that followed a rehabilitation process previously answered use intention and usability for rehabilitation items

Figure 14 shows volunteers’ quantitative answers to the questionnaires on the usability and performance of the VC. Table 11 presents a set of descriptive statistics summarizing quantitative results and Pearson Correlation between dimensions.

**Table 11:** Descriptive statistics and Pearson correlation

	H	U	IU	SP
H	1	0.03	1.00	0.53
U	0.03	1	1.00	0.75
IU	1.00	1.000	1	1.00
SP	0.53	0.75	1.00	1
*Minimum*	3.75	4.00	4.00	3.00
*Maximum*	5.00	5.00	5.00	5.00
*Mean*	4.54	4.86	4.75	4.36
*SD*	0.51	0.38	0.50	0.80

*SD*-standard deviation

From Fig. 14, concerning Hedonic (H) value ($mean = 4.54 \pm 0.51$), most volunteers enjoyed exercising with the VC, felt motivated and interested in the exercises, and found the established interaction pleasant. The most appreciated and motivating features of the system were the “posture corrections” (V01) and the “User Interface” (V05).

Regarding the Utilitarian (U) value, Fig. 14 shows volunteers find the system valuable for post-stroke rehabilitation. Volunteers reported the system ($mean = 4.86 \pm 0.38$):

May be useful for autonomy in exercise practice. (V02)

It can help to motivate the correct exercise performance. (V03)

Concerning Use intention (IU) ($4.75 \pm 0.50$), volunteers, in the case of need, revealed interest in using the system (Figure 14). A volunteer mentioned that he would use the system to “practice more” (V01), enhancing recovery.

Volunteers perceived that the system performs properly and fulfills its purpose. They expressed system’s evaluation of their motor performance was trustworthy. A mean score of 4.36 on the System’s Performance (SP) supports these affirmations. Volunteers revealed:

System proposed corrections matched the movement. (V05)

Reliable, it asks to repeat the exercise and to be perfected. (V02)

However, volunteers provided comments on aspects that need to be improved, such as the VC response time (V06) and more flexibility regarding users’ initial position:

The square that detected my body could be a little bigger because, when moving, the body could leave the square and it was necessary to repeat the exercise. (V07)

#### Virtual coach performance and Hedonic value

We compute the Pearson Correlation coefficient ($\rho$) to analyze the correlation between each dimension (*H*, *U*, *SP*, and *IU*) based on questionnaires quantitative answers (Table 11).

Table 11 shows a correlation between *H* and *SP* with a coefficient of $\rho = 0.53$, revealing that these dimensions are moderately correlated [29]. If the mean value of the perceived *SP* increases, it positively influences the perceived *H*. A volunteer that mentioned “the system has a slow response” also mentioned this aspect when he was asked for the most/least pleasant or interesting system features:

Slow responsive system and interaction could be more stimulating for the participant. (V06)

#### Stroke survivor vs. other volunteers

**Table 12:** Stroke survivor vs. other volunteers mean perceptions

	H	U	IU	SP
Stroke survivor	4.50	4.0	4.0	3.0
Other volunteers	$4.54 \pm 0.56$	$5.0 \pm 0.0$	$5.0 \pm 0.0$	$4.58 \pm 0.58$

We compared the post-stroke survivor’s perceptions with other volunteers’ mean perceptions, shown in Table 12. Concerning *H* perception, the stroke survivor and other volunteers equally enjoyed the training and interact with the system ($mean \approx 4.5$). However, stroke survivor reported a lower mean score for *U* ($mean = 4$) and *SP* ($mean = 3$) and showing a less IU ($mean = 4$):

Certain corrections might be tricky to apply alone. (V01)

#### Age and utilitarian value

Additionally, we analyze how volunteers from different age groups perceive VC utilitarian value *U*. Table 13 shows the mean perception of two age groups: older adults, volunteers over 54 years old ($n = 4$), and the remaining volunteers we consider as younger adults ($n = 3$). Older adults found the system more useful (Table 13). However, despite the mean score difference between groups is 0.3333, this difference is not statistically significant.

**Table 13:** Older and younger adults mean perceptions

Age	*n*	H	U	IU	SP
25–34	3	$4.25 \pm 0.43$	$4.67 \pm 0.58$	$4.0 \pm 0.0$	$4.0 \pm 0.87$
55–64	2	$4.5 \pm 0.71$	$5.0 \pm 0.0$	$5.0 \pm 0.0$	$4.25 \pm 1.1$
65–74	2	$5.0 \pm 0.0$	$5.0 \pm 0.0$	$5.0\pm 0.0$	$5.0 \pm 0.0$
$< 55$	3	$4.25 \pm 0.43$	**4.67** ± **0.58**	$4.0 \pm 0.0$	$4.0 \pm 0.87$
$\ge 55$	4	$4.75 \pm 0.5$	**5.0** ± **0.0**	$5.0 \pm 0.0$	$4.63 \pm 0.75$

The results in bold correspond to the best classifiers' performance for the different metrics for each exercise

## Discussion

### Compensation assessment methods analysis

Table 9 describes the results of RB and NN-based proposed classification approaches. From LOSO cross-validation for each exercise (E1, E2, and E3), we found our methods achieved comparable performance (72–79%) to the models with 3D pose data (74–82%) [20], giving evidence that assessing compensation patterns from 2D pose data is feasible. For E1, the RB approach performs better than NN-based, and for E2 and E3, the NN-based approach presents better results than RB. An evident difference between the datasets of these exercises is their percentage of multi-labeled samples, $1-P_{min}$. E1 has $16.17 \%$ of multi-labeled samples. E2 and E3 have $8.6 \%$ and $1.85 \%$, respectively, of samples with more than one label. This fact implies that the RB method handles multi-labeled samples better than NN-based. On the other hand, the NN-based approach is more efficient than RB with binary problems. For E3, the NN-based approach performs better. However, it has a higher value of *HammingLoss*, meaning that this approach provides a higher number of mispredictions.

Additionally, standard deviation values (Table 9) are related to poor representation of some compensation patterns in the dataset. The RB and the NN-based classifiers reveal an average standard deviation of $18\%$ and $21.7\%$ in $F_1$, respectively, for the three exercises. These standard deviation values, associated with the adopted validation method, LOSO, indicate that our classifiers detect with higher accuracy some compensation patterns than others. The NN-based approach, which involves learning, has more difficulty identifying rarer compensation patterns in the dataset. This approach would benefit from more data with a homogenous representation of the different compensation patterns. However, we consider our results for the $F_1$ score comparable to the agreement level of annotators (i.e., $79.08 \pm 21.46\%$ for E1, $82.22 \pm 15.34\%$ for E2, and $71.96 \pm 17.54\%$ for E3) [20]. Personalized assessment techniques can improve performance evaluation from patient to patient, as in [20]. These techniques promote the generation of personalized quality of movement evaluation and corrective feedback in opposition to pre-defined rules and threshold values, which might not fit properly every patient.

Results from LOEO cross-validation for the NN-based approach ($F_1$ score of $79.59\% \pm 1.86\%$) show us that the models can generalize to other exercises as long as the setup for data collection is the same (i.e., patients’ position in front of the camera).

### Virtual coach experiment analysis

From the exploratory experiment with a group of volunteers, we collected a set of findings on VC usability and performance. Quantitative scores on each dimension perceptions (Fig. 14) and volunteers’ quotes show the low-cost VC has the potential to automatically monitor participants’ exercises and provide valuable feedback on compensatory motions. In general, volunteers enjoyed the exercise session with the VC, found it beneficial, and its movement analysis trustworthy.

By analyzing the impact of System Performance on volunteers perception of Hedonic Value (***H1***), we found some points requiring improvement: lack of flexibility concerning volunteers initial position; the slow response of the system to users’ movements; and motion pattern mispredictions.

Volunteer V07 (Table 8) referred system’s lack of flexibility with her initial position as an unappreciated feature, negatively affecting her interest and enjoyment in the activity. In some sessions, due to space conditions, we were unable to assure subjects correct positioning to place one’s body inside the rectangle. For this reason, the system assumed the subject was incorrectly positioned to perform movement assessment.

Volunteer V06 (Table 8) pointed out the system’s slow response to his movements. In some cases, the system had a slower response when providing volunteers with feedback on their movements due to internet connection conditions since main processing steps occur in a remote server accessed through WiFi.

Additionally, during the study, we detected unexpected compensation mispredictions (RB approach). In some cases, when a user tilted the torso, the VC assumed the user was performing shoulder elevation since it detected shoulder angular displacement. This VC behavior suggests a review in rule implementation and an improvement of the RB approach to avoid the detection of shoulder compensation while a trunk compensatory movement occurs.

When comparing stroke survivor and other volunteers’ perceptions (***H2.a***), results reveal stroke survivor was more critical with the system than other volunteers (Table 12). The stroke survivor commented on compensation detection sensitivity. In his opinion, the VC should not be too sensitive, i.e., give feedback on compensation immediately when a patient is just beginning to perform a compensatory movement, thus very pronounced yet. It should provide patients with time and opportunity to perform the proposed movements and improve themselves without being constantly and immediately corrected.

In our population sample, older adults find the VC more useful (U) than younger adults (***H2.b***). This difference is expected since stroke is more prevalent among older adults and the elderly. However, the mean difference between both groups in U perception difference is only 0.3333, and the independent sample t-test revealed it to be insignificant. It is important to note that both groups have small and unequal number of subjects (young volunteers $n = 3$; older volunteers $n = 4$), *n*, a condition that can lead to an untrustworthy $p-value$. To collect more significant results, we would need to conduct a user study with a larger group of volunteers and a homogeneous distribution of age categories.

### Limitations and future work

To continue the investigation of motor compensation detection methods from 2D positional data, we aim to explore other assessment approaches and machine learning models. Our RB approach could be improved to avoid the detection of compensation patterns involving shoulder angular/linear displacement when trunk compensation occurs. Priority could be given to trunk displacements over shoulder elevation patterns to overcome some misdetections. Additionally, we intend to expand VC’s quality of movement assessment to other performance components, such as Range-of-Motion and Smoothness [9]. Further, we aim to achieve the generation of a performance score with clinical relevance, as in [9]. It would provide patients with exercise ratings promoting motivation and give therapists significant information to track patients’ progress.

Another relevant improvement of our VC is its response time to users’ movements (e.g., track arm movements and detect compensation), which is directly related to the connection via WiFi to the remote server in which main processing steps occur. Previous works [30] propose an architecture for a cognitive wearable assistive system that resorts to remote processing, having achieved faster response time. Additionally, to achieve faster processing, we might benefit from available frameworks, as TensorFlow Lite, and hardware accelerators for AI computing, such as Google CORAL and Inter Neural Compute Stick 2.

We could give therapists the possibility to adjust the threshold values that control the RB method rules [31], managing compensation detection sensitivity through the UI. It could enable exercise level adaptation based on compensation detection sensitivity (more sensitive, more challenging).

Additionally, once we have improved the VC according to the findings achieved in this study, the VC should be evaluated with post-stroke patients under a rehabilitation process and therapists.

## Conclusions

This work contributes to the research of assistive systems for in-home rehabilitation. With the dataset of 15 post-stroke patients, we demonstrate that the proposed methods accurately assess motor compensation from 2D positional data. The proposed low-cost motion analysis approach using 2D videos can achieve comparable performance with compensatory motion assessment approaches using 3D pose data [20]. In addition, during the preliminary user study with a group of volunteers, as desired the VC provides helpful visual and audio feedback and accurately tracks users’ movements. Additionally, we identified some points for improvement and collected evidence towards the feasibility of the low-cost virtual coach (VC) for stroke rehabilitation.

## Data Availability

The datasets and additional data gathered and/or analyzed in this study are not publicly available due to population vulnerability (i.e., patients under a post-stroke rehabilitation process) and the personal nature of video recordings captured in a hospital and domestic environments.

## Abbreviations

2D: Two-dimensional
3D: Three-dimensional
FMA: Fugl-Meyer assessment
WMFT: Wolf Motor function test
RB: Rule-based
MLC: Multilabel classification
MLD: Multilabel dataset
NN: Neural network
UI: User interface
VC: Virtual coach
